# Supplementary figures and images for: Myc 9aaTAD activation domain binds to mediator of transcription with superior high affinity
Source: Mol Med. 2024 Nov 13;30:211. doi: 10.1186/s10020-024-00896-7 (PMC11558822; doi:10.1186/s10020-024-00896-7)

Suppl. Figure S1

Westernblotting:  
(anti-HA antibody)

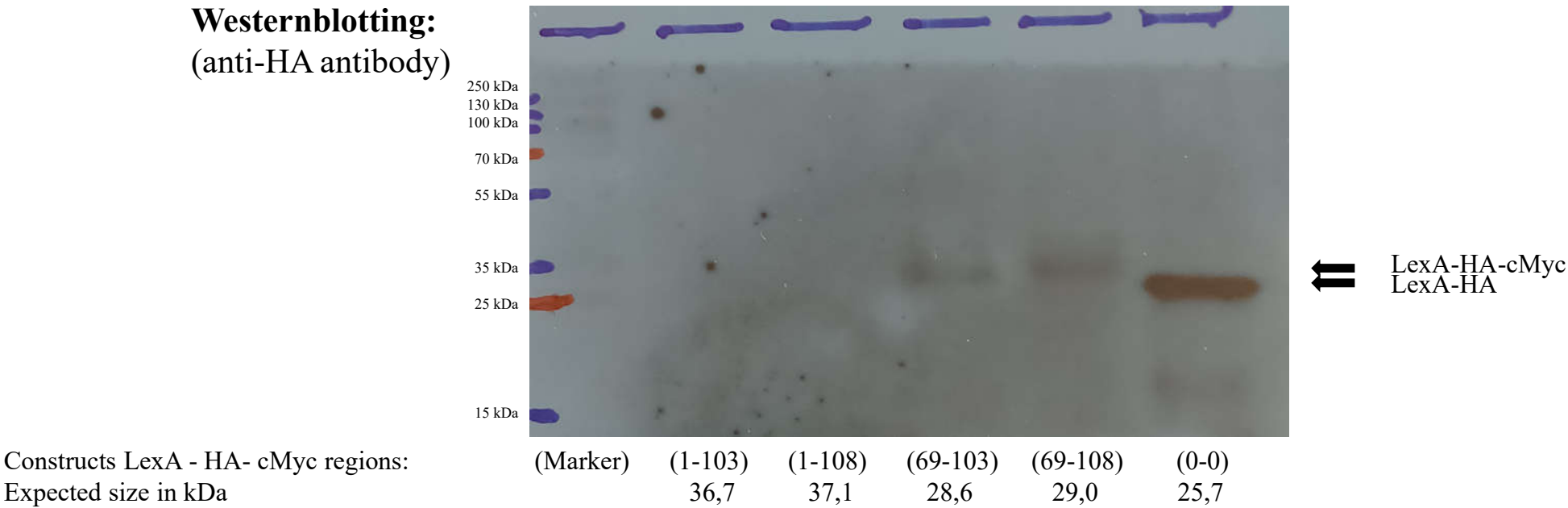

Westernblotting - repeat:  
(anti-HA antibody)

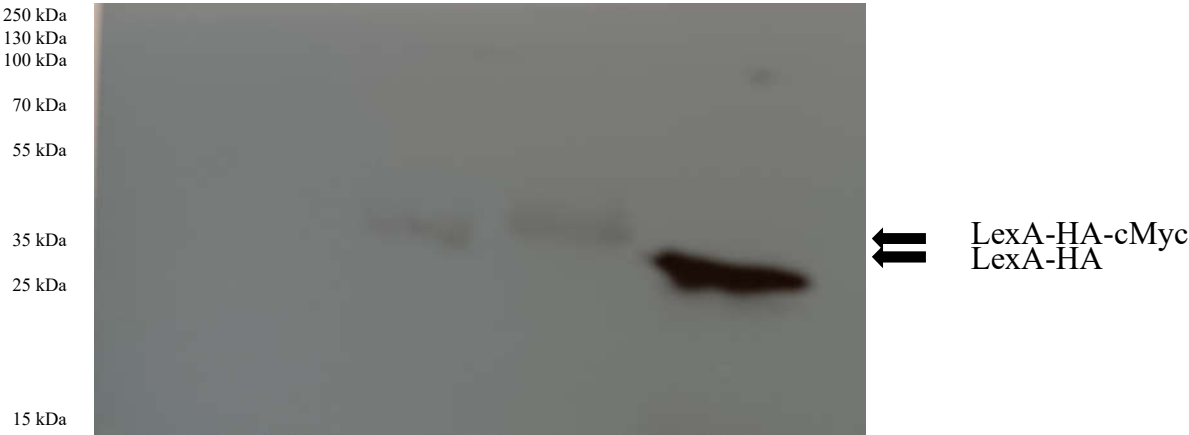

Supplement: Supplementary file 1 — Additional file 1. [file 10020_2024_896_MOESM1_ESM.pdf]
