# Supplementary material for: Myc 9aaTAD activation domain binds to mediator of transcription with superior high affinity
Source: Mol Med. 2024 Nov 13;30:211. doi: 10.1186/s10020-024-00896-7 (PMC11558822; doi:10.1186/s10020-024-00896-7)
Supplement: Supplementary file 2 — Additional file 2. [file 10020_2024_896_MOESM2_ESM.pdf]

Sample		time in min	time in second	A420	Protein	ß-Gal	normalized value in %	average value in %	SD
**1-103**	1	35	2100	9	6,49	25	9	9	1
	2	35	2100	9	6,84	24	9		
	3	35	2100	10	6,72	27	10		
**1-108**	4	35	2100	51	6,79	136	50	49	3
	5	35	2100	44	6,45	123	45		
	6	35	2100	50	6,50	139	51		
**69-103**	7	35	2100	28	6,84	74	27	29	2
	8	35	2100	32	6,95	83	31		
	9	35	2100	26	6,63	71	26		
**69-108**	10	35	2100	95	6,75	255	93	90	3
	11	35	2100	87	6,52	241	88		
	12	35	2100	92	6,88	242	89		
**98-108**	13	35	2100	80	7,56	191	70	67	4
	14	35	2100	82	8,02	185	68		
	15	35	2100	76	8,12	169	62		
**empty**	16	35	2100	0	6,61	0	0	0	1
	17	35	2100	1	6,79	3	1		
	18	35	2100	0	6,84	0	0		
**6p53**	19	20	1200	52	6,15	**268**	98	100	3
	20	20	1200	55	6,18	**282**	103		
	21	20	1200	59	6,93	**270**	99		
						**273**			
